# Supplementary material for: Unlocking ultra-high holographic information capacity through nonorthogonal polarization multiplexing
Source: Nat Commun. 2024 Jul 26;15:6284. doi: 10.1038/s41467-024-50586-5 (PMC11282074; doi:10.1038/s41467-024-50586-5)
Supplement: Supplementary file 1 — Supplementary Information [file 41467_2024_50586_MOESM1_ESM.pdf]

Supplementary Materials for:

Unlocking Ultra-High Holographic Information Capacity through Nonorthogonal Polarization Multiplexing

Jie Wang[1,2#], Jin Chen[1#], Feilong Yu[1#], Rongsheng Chen[1], Jiuxu Wang[1], Zengyue Zhao[1], Xuenan Li[1], Huaizhong Xing[2], Guanhai Li[1,3,4,5*], Xiaoshuang Chen[1,3,4,5], Wei Lu[1,3,4,5]

[1]State Key Laboratory of Infrared Physics, Shanghai Institute of Technical Physics, Chinese Academy of Sciences, 500 Yu-Tian Road, Shanghai, 200083, China

[2]DongHua University, 2999 North Renmin Road, Shanghai 201620, China

[3]Hangzhou Institute for Advanced Study, University of Chinese Academy of Sciences, No.1 SubLane Xiangshan, Hangzhou, 310024, China

[4]University of Chinese Academy of Science, No. 19 Yuquan Road, Beijing, 100049, China

[5]Shanghai Research Center for Quantum Sciences, 99 Xiupu Road, Shanghai, 201315, China

#These authors contributed equally: Jie Wang, Jin Chen, Feilong Yu.

*ghli0120@mail.sitp.ac.cn

Note 1. Derivation of upper-limit of asymmetric channels in a single metasurface

In our study, we explore the derivation of the upper limit of asymmetric channels in a single metasurface, focusing on the Jones matrix representation of forward propagation. Specifically, J_b defines the transmission matrix for light traversing the structure, rotated by 180° relative to the x-axis (with the choice of x or y axis being arbitrary).

Considering only reciprocal media, the Jones matrix can be expressed as:

$$J_b = \begin{bmatrix} A & -C \\ -B & D \end{bmatrix} \qquad (s1)$$

If the metaatom exhibits mirror symmetry with respect to the xz plane, the Jones matrix for the structure reflected in this plane remains identical to the original. Consequently, we obtain:

$$M_x = \begin{bmatrix} 1 & 0 \\ 0 & -1 \end{bmatrix}, \quad M_x^{-1} \cdot J \cdot M_x = \begin{bmatrix} A & -B \\ -C & D \end{bmatrix} = J$$
$$\rightarrow J = \begin{bmatrix} A & 0 \\ 0 & D \end{bmatrix} \qquad (s2)$$

where M_x is the reflection matrix with respect to the x-axis. Thus, any structure with this symmetry can effectively be described by a diagonal Jones matrix.

Similarly, if the metaatom is mirror-symmetric with respect to the yz plane, we find:

$$M_y = \begin{bmatrix} -1 & 0 \\ 0 & 1 \end{bmatrix}, \quad M_y^{-1} \cdot J \cdot M_y = \begin{bmatrix} A & -B \\ -C & D \end{bmatrix} = J$$
$$\rightarrow J = \begin{bmatrix} A & 0 \\ 0 & D \end{bmatrix} \qquad (s3)$$

Hence, for any structure with a mirror plane parallel to the z-axis, the transmission matrix is diagonal, assuming the mirror plane aligns with the x or y axes. In such systems, the eigenstates of polarization are inherently linear.

Further investigation into potential mirror symmetries perpendicular to the z-axis reveals that for structures possessing this symmetry, the reflected structure mirrors the rear view:

$$M_x^{-1} \cdot J \cdot M_x = \begin{bmatrix} A & -B \\ -C & D \end{bmatrix} = \begin{bmatrix} A & -C \\ -B & D \end{bmatrix} = J_b \\ \rightarrow J = \begin{bmatrix} A & B \\ B & D \end{bmatrix} \tag{s4}$$

Adopting $\boldsymbol{p_j} = \begin{bmatrix} cos\alpha_j \\ sin\alpha_j \cdot e^{i\beta_j} \end{bmatrix}$ and $\boldsymbol{p_k} = \begin{bmatrix} \cos\alpha_k \\ \sin\alpha_k\, e^{i\beta_k} \end{bmatrix}$ from the main text **Eq. 2**, we derive:

$$O_{jk}(x,y) = \langle p_k|J|p_j\rangle = \begin{bmatrix} \cos\alpha_k \\ \sin\alpha_k\, e^{i\beta_k} \end{bmatrix}^{\dagger} \cdot \begin{bmatrix} A & B \\ B & D \end{bmatrix} \cdot \begin{bmatrix} \cos\alpha_j \\ \sin\alpha_j\, e^{i\beta_j} \end{bmatrix} \\ = \cos\alpha_j \cos\alpha_k\, A + \sin\alpha_j \sin\alpha_k\, e^{i(\beta_j-\beta_k)} D \\ +\left(\sin\alpha_j \cos\alpha_k\, e^{i\beta_j} + \cos\alpha_j \sin\alpha_k\, e^{-i\beta_k}\right) B \tag{s5}$$

This leads to the interchange of input and output polarizations in directional transmission:

$$O_{kj}(x,y) = \cos\alpha_j \cos\alpha_k\, A + \sin\alpha_j \sin\alpha_k\, e^{-i(\beta_j-\beta_k)} D \\ +\left(\sin\alpha_j \cos\alpha_k\, e^{-i\beta_j} + \cos\alpha_j \sin\alpha_k\, e^{i\beta_k}\right) B \tag{s6}$$

For linearly polarized light, β vanishes, and $O_{jk}(x,y) = O_{kj}(x,y)$. The Jones matrix becomes symmetric:

$$\boldsymbol{O}(x,y) = \begin{bmatrix} O_{11}(x,y) & O_{12}(x,y) & O_{13}(x,y) & \dots & O_{1n}(x,y) \\ O_{12}(x,y) & O_{22}(x,y) & O_{23}(x,y) & \dots & O_{2n}(x,y) \\ O_{13}(x,y) & O_{23}(x,y) & O_{33}(x,y) & \dots & O_{3n}(x,y) \\ \vdots & \vdots & \vdots & \ddots & \vdots \\ O_{1n}(x,y) & O_{2n}(x,y) & O_{3n}(x,y) & \dots & O_{nn}(x,y) \end{bmatrix} \tag{s7}$$

Due to the reciprocity of the metasurface, the polarization multiplexing channels O_{jk} and O_{kj} are the same. Therefore, the maximum number of channels is represented by the upper (or lower) nonzero triangular matrix, as shown in the main text **Eq. 4**. In this formula, n (n=1,2,3,4,5,6…) refers to the number of pairs of input and output polarization states. Our findings indicate that previous research has not achieved this limit. Specifically, for symmetric channels (*j*=*k*), polarization introduces degrees of freedom that modulate only the amplitudes of the Jones matrix elements (A, B, D). The paper Ref. 17 is the case. Extending to asymmetric channels introduces both amplitude and phase modulation, vastly enhancing our control capabilities.

Note 2. Establishment of the vectorial diffraction neural network

In our endeavor to refine the design of holographic patterns with varying polarizations, we have synergized the principles of polarization decomposition theory, optical diffraction theory, and neural network architectures. By leveraging the vectorial data extracted from optical wavefronts and utilizing the advanced pattern recognition and learning capabilities inherent in neural networks, we have developed the vectorial diffraction neural network (VDNN). This network demonstrates exceptional adaptability to mode variations in diverse optical environments and under fluctuating input conditions. The metasurface, designed with this approach, exhibits intelligent and efficient information processing in dynamic optical scenarios, moving beyond the limitations of orthogonal polarization constraints. This advancement paves the way for the practical realization of non-orthogonal polarization multiplexing in holography.

The neural network model operates via forward propagation, which involves the modulation of metaatoms and the subsequent optical diffraction across various layers. According to Eq. 1, each metaatom can be represented by a Jones matrix with distinct eigen-polarization and eigen values. This concept is analogous to the trainable parameters (weights and biases) in neural networks, which determine how input data is transformed into output data. By mapping the nodes in a neural network to the response of the metaatoms $O_{\mathrm{ij}}(x, y)$ in Eq. 2, we can optimize and obtain the desired local eigen-polarization. 55 nonorthogonal polarization channels can be trained in parallel within the network with the form of Eq. 4 at a global level. The specific derivation process is as follows.

This modulation in the neural network enables the comprehensive representation of any incident light, E_{in}, which can be mathematically expressed as $\boldsymbol{E}_{in} = \begin{bmatrix} \cos\alpha_1 \\ \sin\alpha_1 \cdot e^{i\beta_1} \end{bmatrix}$. Eq. s8 illustrates the capacity of our system to transcribe and manipulate incident light in a versatile manner, thereby optimizing the holographic output for various applications and conditions.

$$\boldsymbol{E}_{out} = \begin{bmatrix} E_{ox} \\ E_{oy} \end{bmatrix} = T * E_{in} = \begin{bmatrix} T_{xx} & T_{yx} \\ T_{xy} & T_{yy} \end{bmatrix} * \begin{bmatrix} \cos\alpha_1 \\ \sin\alpha_1 \cdot e^{i\beta_1} \end{bmatrix}$$
$$= \begin{bmatrix} cos(-\theta) & -sin(-\theta) \\ sin(-\theta) & cos(-\theta) \end{bmatrix} * \begin{bmatrix} a_x e^{i\varphi_x} & 0 \\ 0 & a_y e^{i\varphi_y} \end{bmatrix} * \begin{bmatrix} cos\,\theta & -sin\theta \\ sin\,\theta & cos\,\theta \end{bmatrix} * \begin{bmatrix} \cos\alpha_1 \\ \sin\alpha_1 \cdot e^{i\beta_1} \end{bmatrix} \quad \text{(s8)}$$

where $T_{xy} = T_{yx}, |T_{xx}|^2 = |T_{yy}|^2$. Eq. s8 can be expressed in terms of $E_{out} = M(E_{in}) = T * E_{in}$. $M(*)$ contains the learnable parameters of a_x, φ_x, a_y, φ_y and θ.

$\boldsymbol{E}_{ox}$ and $\boldsymbol{E}_{oy}$ represent the decomposition of polarized light with x- and y- orthogonal bases. In accordance with the Huygens-Fresnel principle, metaatoms on each layer can be treated as independent secondary wave sources. The electric field of (*p*, *q*) metaatom located in layer *l* is transferred to the electric field of the (*m*, *n*) in layer *l+1*:

$$E_{m,n}^{l+1} = w_{p,q}^{l} \cdot M\left(E_{p,q}^{l}\right), \qquad m, n, p, q \subset \left[-\frac{N}{2}, \frac{N}{2}\right] \quad \text{(s9)}$$

In this way, electric field at layer *l+1* is $\boldsymbol{E}^{l+1} = \sum_p \sum_q \left(\boldsymbol{w}^{l} \cdot M\left(\boldsymbol{E}^{l}\right)\right)$, where $\boldsymbol{w}^{l}$ is the tunable hyperparameter and $M(*)$ contains the trainable model parameters, which is different to the traditional neural network.

The training models on different layers are different. The transition from the input to the hidden layer can be expressed as $\boldsymbol{E}^{1} = \sum_p \sum_q \boldsymbol{E}^{0}$, meaning that $\boldsymbol{w}^{l} = \mathbf{1}$ in Eq. s9; while the transitions between different hidden layers is $w_{p,q}^{l} = \frac{e^{-ikr_{p,q}}}{i\lambda r_{p,q}}, r_{p,q} = \sqrt{\left(x - x_{p,q}\right)^2 + \left(y - y_{p,q}\right)^2 + z^2}, k = \frac{2\pi}{\lambda}, i = \sqrt{-1}$. Before the light reaches the output layer, it is modulated by the polarization analyzer $\begin{bmatrix} \cos\alpha_2 \\ \sin\alpha_2 \cdot e^{i\beta_2} \end{bmatrix}$. In this way, the designed hologram with specific polarization is achieved.

Furthermore, we incorporate an error function to facilitate the optimization of the trainable parameters within the function $M(*)$ for the back-propagation algorithm. The introduced loss function is defined using the mean squared error (MSE), a metric commonly employed for assessing image similarity. This function evaluates the performance by comparing the output intensity E_{pq} with the target intensity T_{pq} at each point: $MSE(T, E) = \frac{1}{N^2} \sum_p^N \sum_q^N \left(T_{pq} - E_{pq}\right)^2$. Here, *N* represents the number of atoms on each row or column of the metasurface. The optimization function Adam is selected, with a learning rate set to 0.015. During the backpropagation process, the cumulative

error across multiple outputs and target patterns is fed back into the optimization function. This iterative process updates the weight parameters within the neural network, minimizing the error in the loss function until the network achieves stability.

In the training of three-channel linear polarization multiplexing, we set the transmittance of the metaatoms to 1, meaning that the training parameters only include phase and rotation angle. Given that the designed single-layer metasurface consists of 400 × 400 metaatoms, the total number of node parameters is $3 \times 400 \times 400 = 4.8 \times 10^5$. For the training of three-channel circular and elliptical polarization multiplexing, we include the transmittance of the metaatoms, resulting in a total of $5 \times 400 \times 400 = 8 \times 10^5$ node parameters. In the training of 55-channel multiplexing, there are a total of $5 \times 800 \times 800 = 3.2 \times 10^6$ node parameters involved. Similar calculations apply to other cases in the main text.

Note 3. Database of metaatoms

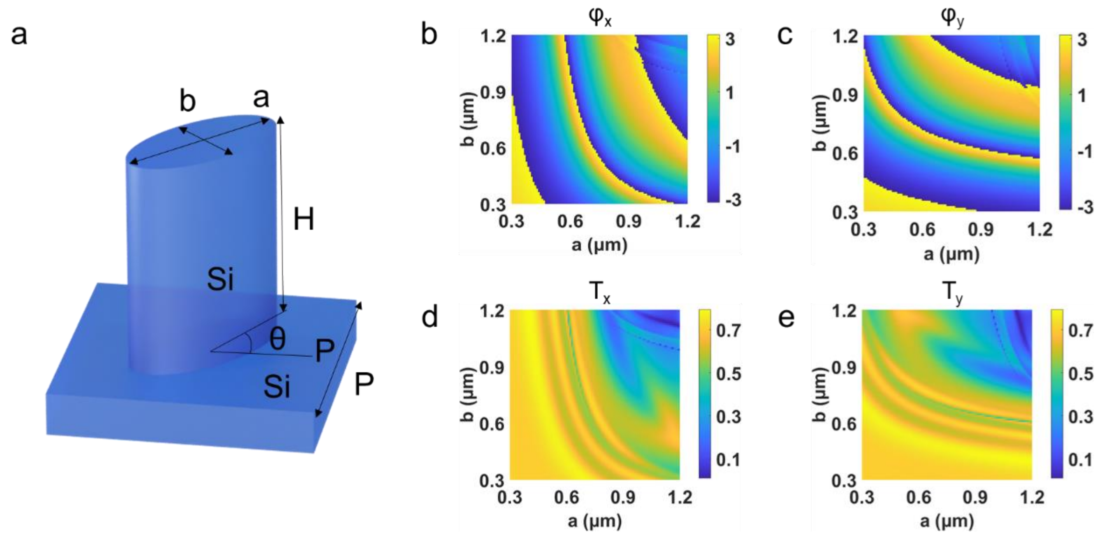

Supplementary Fig. 1. Schematic illustration of a metaatom and the phase and transmittance database. a Three-dimensional diagram of the metaatom used in the metasurface design. **b-c** Phase variation of the metaatom under x- and y-polarization as a function of its length and width. **d-e** Transmittance variation of the metaatom under x- and y-polarization as a function of its length and width.

The metaatoms used in this paper are all elliptic cylindrical structures with different structural parameters. The phase and transmittance data shown in Supplementary Fig. 1 were obtained under incident light with a wavelength of 3 μm. To ensure uniformity in certain designs, we limit the transmittance to maintain consistent performance across the metaatoms.

Note 4. Design degrees of freedom (DoFs) in conventional Jones matrix

In the case of a unitary planar system, characterized by mirror symmetry out-of-plane, crosstalk among cross-polarized channels becomes evident. The Jones matrix representation of such a system is expressed as:

$$J = \begin{bmatrix} A & B \\ C & D \end{bmatrix} = [\widehat{\boldsymbol{e_1}}, \widehat{\boldsymbol{e_2}}]^{-1} \begin{bmatrix} \Lambda_1 & 0 \\ 0 & \Lambda_2 \end{bmatrix} [\widehat{\boldsymbol{e_1}}, \widehat{\boldsymbol{e_2}}] \qquad (s10)$$

Here, $\widehat{\boldsymbol{e_1}}$ and $\widehat{\boldsymbol{e_2}}$ represent the eigenpolarizations, and Λ_1 and Λ_2 are the corresponding eigenvalues. This formulation reveals that a single-layer metasurface provides a maximum of 3 degrees of freedom for modulation. The modulation channel is inherently constrained by the eigenvectors of the system.

For an arbitrary incident polarization $\boldsymbol{p_j} = \begin{bmatrix} cos\alpha_j \\ sin\alpha_j \cdot e^{i\beta_j} \end{bmatrix}$ and analyzing polarization $\boldsymbol{p_k} = \begin{bmatrix} \cos\alpha_k \\ \sin\alpha_k \cdot e^{i\beta_k} \end{bmatrix}$($j$, k = 1, 2, …, n), the transmitted light can be written as

$$E_{out}(\alpha_{jk}, \beta_{jk}) = \begin{bmatrix} \cos\alpha_k \\ \sin\alpha_k \cdot e^{i\beta_k} \end{bmatrix}^{\dagger} \cdot \begin{bmatrix} a_A e^{i\varphi_A} & a_B e^{i\varphi_B} \\ a_B e^{i\varphi_B} & a_D e^{i\varphi_D} \end{bmatrix} \cdot \begin{bmatrix} \cos\alpha_j \\ \sin\alpha_j \cdot e^{i\beta_j} \end{bmatrix}$$
$$= \begin{bmatrix} \cos\alpha_j \cdot \cos\alpha_k & \cos\alpha_j \cdot \sin\alpha_k \cdot e^{-i\beta_k} + \sin\alpha_j \cdot \cos\alpha_k \cdot e^{i\beta_j} & \sin\alpha_j \cdot \sin\alpha_k \cdot e^{i(\beta_j - \beta_k)} \end{bmatrix} \cdot \begin{bmatrix} a_A e^{i\varphi_A} \\ a_B e^{i\varphi_B} \\ a_D e^{i\varphi_D} \end{bmatrix} (s11)$$

For three cyclic nonorthogonal input and output polarizations (j = 1, 2, 3, k = 2, 3, 1), the expression becomes:

$$\begin{bmatrix} O_1 \\ O_2 \\ O_3 \end{bmatrix} = \begin{bmatrix} \cos\alpha_1 \cdot \cos\alpha_2 & \cos\alpha_1 \cdot \sin\alpha_2 \cdot e^{-i\beta_2} + \sin\alpha_1 \cdot \cos\alpha_2 \cdot e^{i\beta_1} & \sin\alpha_1 \cdot \sin\alpha_2 \cdot e^{i(\beta_1 - \beta_2)} \\ \cos\alpha_2 \cdot \cos\alpha_3 & \cos\alpha_2 \cdot \sin\alpha_3 \cdot e^{-i\beta_3} + \sin\alpha_2 \cdot \cos\alpha_3 \cdot e^{i\beta_2} & \sin\alpha_2 \cdot \sin\alpha_3 \cdot e^{i(\beta_2 - \beta_3)} \\ \cos\alpha_3 \cdot \cos\alpha_1 & \cos\alpha_3 \cdot \sin\alpha_1 \cdot e^{-i\beta_1} + \sin\alpha_3 \cdot \cos\alpha_1 \cdot e^{i\beta_3} & \sin\alpha_3 \cdot \sin\alpha_1 \cdot e^{i(\beta_3 - \beta_1)} \end{bmatrix} \cdot \begin{bmatrix} a_A e^{i\varphi_A} \\ a_B e^{i\varphi_B} \\ a_D e^{i\varphi_D} \end{bmatrix} (s12)$$

In this linear equation, $\boldsymbol{O} = \boldsymbol{KX}$ represents the hologram, $\boldsymbol{K}$ signifies the coefficient matrix, and $\boldsymbol{X}$ is the Jones matrix. The matrix is treated as three variables in this context, representing the response of the metaatom.

Given that the rank of the coefficient matrix K is three, a unique solution for $\boldsymbol{O} = \boldsymbol{KX}$ exists. This implies the presence of three independent channels. To validate this, a sampling method is employed for verification. Taking linear polarization as an example,

with the polarization angle α selected at 1-degree intervals, and considering the three variables in the equations, the matrix's rank needs to be calculated 180×180×180 = 5,832,000 times. After accounting for the distinct polarizations, 180×179×178 = 5,735,160 cases remain. Numerical analysis consistently reveals that the rank of the coefficient matrix is always 3 in all cases, extending to any elliptic polarization, including circular polarization. In order to represent the full coverage of the polarization states in the main text, we choose the input and output polarization which follows a circular rule. Actually, there is no specific requirement for the choice of operation polarization states.

Note 5. Evaluation of crosstalk of the generated holographic images

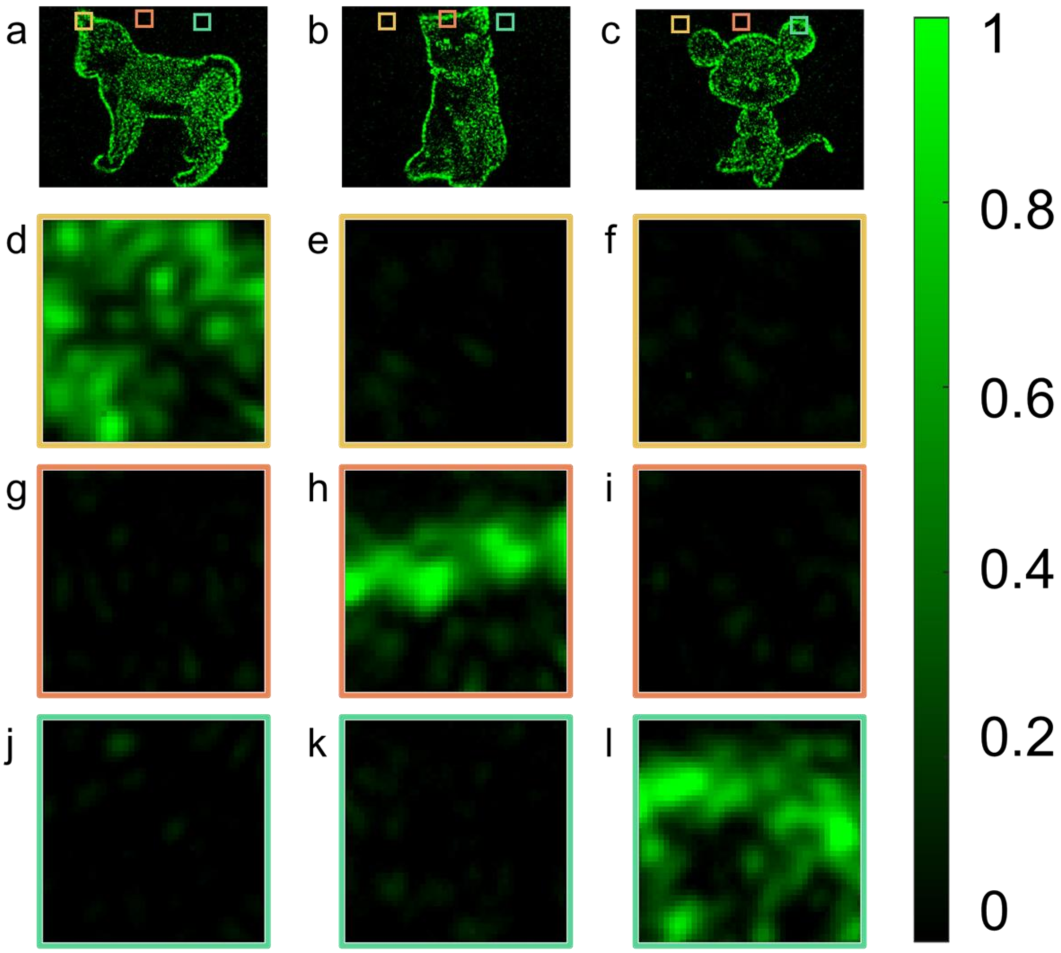

Supplementary Fig. 2. Evaluation of crosstalk of the generated holographic images. a-c Experimental results of the holograms and the selected areas for crosstalk evaluation. Three distinct regions are highlighted by yellow, orange, and green boxes in the experimental images, serving as sampling positions for crosstalk assessments. **d-l** Zoomed-in view of the selected areas. The border color of the horizontal row of images corresponds to the enlarged area of the corresponding color in a-c. The images in each vertical column correspond to the enlarged area of the corresponding color in the experimental results above.

a

Average

40×40	Area 1 Yellow	Area 2 Orange	Area 3 Green
Puppy	0.3367	0.0108	0.0118
Kitten	0.0117	0.2354	0.0040
Mouse	0.0090	0.0147	0.3208

b

Normalization

40×40	Area 1 Yellow	Area 2 Orange	Area 3 Green
Puppy	1.000	0.0321	0.0351
Kitten	0.0496	1.000	0.0170
Mouse	0.0282	0.0458	1.000

c

Average

60×60	Area 1 Yellow	Area 2 Orange	Area 3 Green
Puppy	0.2492	0.0080	0.0110
Kitten	0.0110	0.3138	0.0130
Mouse	0.0081	0.0121	0.2137

d

Normalization

60×60	Area 1 Yellow	Area 2 Orange	Area 3 Green
Puppy	1.000	0.0320	0.0440
Kitten	0.0350	1.000	0.0413
Mouse	0.0379	0.0565	1.000

Supplementary Fig. 3. Average and normalization of regional sum under different random sampling sizes. a-b Results obtained with a random sampling size of 40×40 pixels. The figures depict the average and normalization of regional sums, providing insights into the characteristics of the data at this specific sampling scale. **c-d** Corresponding results for a larger sampling size of 60×60 pixels. These figures offer a comparative analysis, showcasing the changes in the average and normalization of regional sums with the alteration in the sampling size.

In the experiments on linear polarization multiplexing, as depicted in Supplementary Fig. 2, three distinct regions were randomly selected, each comprising 40×40 pixels, to assess crosstalk. This choice was made because the three holographic images were captured within the same imaging area. Subsequently, the sum of pixel values within each region was computed individually, and normalization was applied to the corresponding regions in different images. Enlarged views of these selected disjoint regions are illustrated in Supplementary Figs. 2d-l.

To further investigate the impact of different sampling sizes (40×40 and 60×60 pixels), we calculated the average pixel values and normalized results, as presented in Supplementary Fig. 3. The outcomes reveal that our nonorthogonal design scheme effectively mitigates crosstalk between distinct channels, demonstrating a robust suppression mechanism.

Note 6. The multiplexing of arbitrarily nonorthogonal linear polarizations

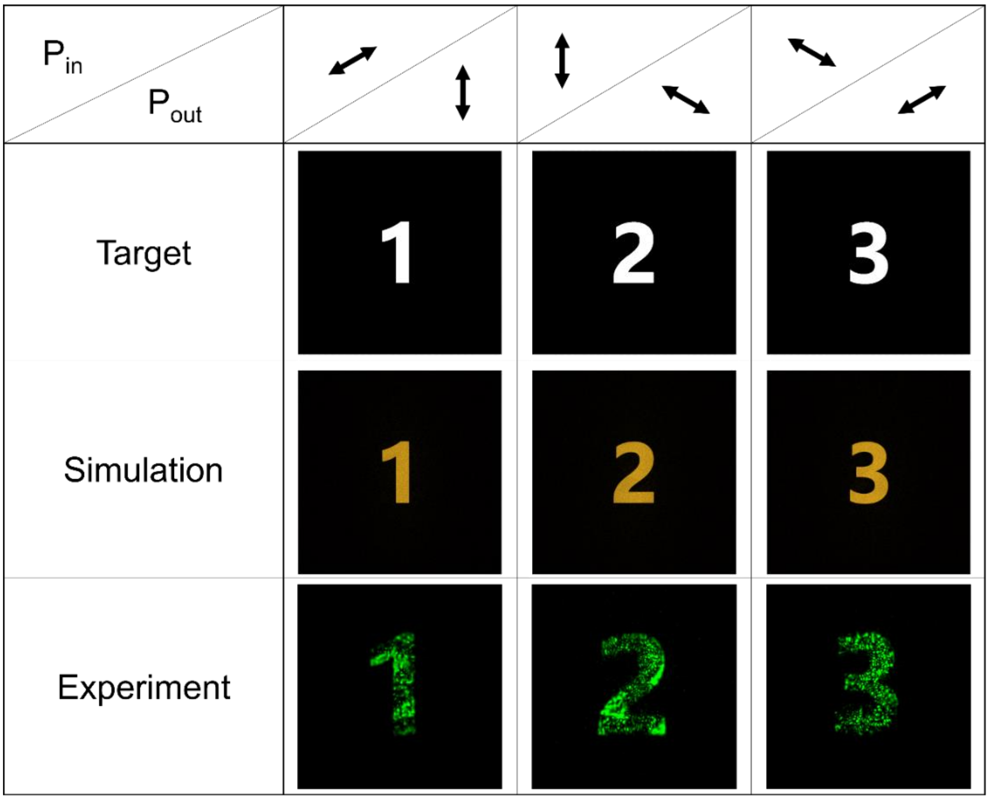

Supplementary Fig. 4. Targets, simulation, and experimental results for three different nonorthogonal linear polarization states (35, 85, 140 degrees) multiplexing. It provides a comprehensive visual representation of targets, simulation outcomes, and experimental results pertaining to three distinct nonorthogonal linear polarization states—specifically, at 35, 85, and 140 degrees. The images collectively offer insights into the efficacy and performance of the multiplexing approach across different polarization angles.

To illustrate nonorthogonal linear polarization multiplexing, we have selected three linear polarizations at 35, 85, and 140 degrees. The simulation and experimental results are presented in Supplementary Fig. 4, corresponding to the following three channels: 1. Incident polarization at 35 degrees, output polarization at 85 degrees, resulting in the output image depicting the number '1'. 2. Incident polarization at 85 degrees, output polarization at 140 degrees, leading to the output image displaying the number '2'. 3. Incident polarization at 140 degrees, output polarization at 35 degrees, resulting in the output image featuring the number '3'. This representation effectively demonstrates the encoding and decoding of information through nonorthogonal linear polarizations, showcasing the multiplexing capabilities of the proposed scheme.

Note 7. Design principle and experimental results of the nine-channel multiplexing

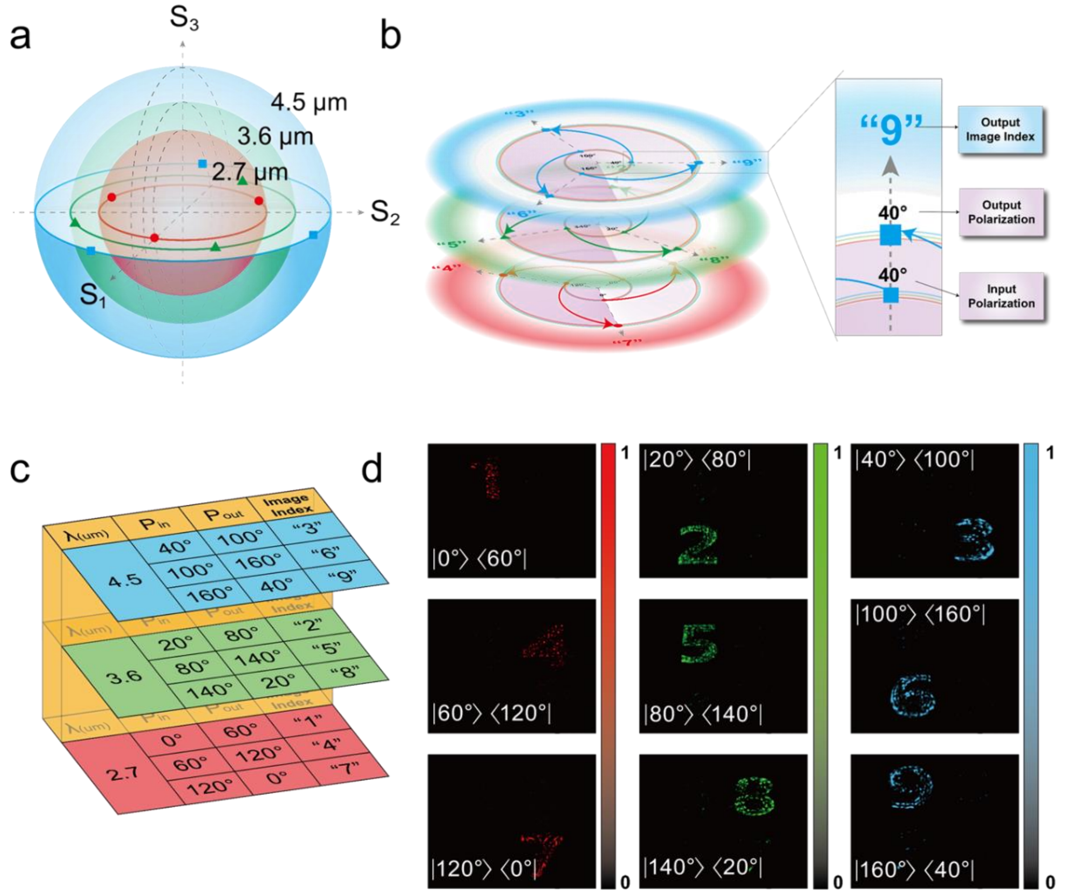

Supplementary Fig. 5. Design principle and experimental results of the nine-channel nonorthogonal polarization multiplexing. a The Poincaré sphere representation illustrating the wavelength and polarization characteristics of the nine-channel multiplexing. **b** A schematic figure depicting the nine-channel polarization conversion process and the corresponding output image serial numbers. **c** A table detailing the wavelength and polarization conversions based on the diagram in (b). **d** Experimental results of the nine-channel nonorthogonal polarization multiplexing. The Dirac symbol on the left signifies the incident polarization, while the symbol on the right indicates the analyzer polarization.

We have extended the nonorthogonal polarization-bases multiplexing approach to accommodate nine channels across three wavelengths and three polarizations. In this expanded scheme, the amplitude, phase, and orientation of metaatoms are incorporated as training parameters. It is crucial to highlight that the orientation of metaatoms remains consistent across all channels, as each metaatom functions universally for every channel. In Supplementary Fig. 5a, we illustrate the distribution of wavelengths

and polarizations on a Poincaré sphere. Different radii are utilized to represent wavelengths of 2.7, 3.6, and 4.5 μm, while distinct shapes (circles, triangles, and squares) indicate the polarization distribution. Supplementary Fig. 5b delineates the polarization channels, with different layers corresponding to operation wavelengths. Within each layer, the inner circle represents the incident polarization, the outer circle indicates the analyzer polarization, and the outermost circle denotes the output image. A comprehensive listing of all channels is presented in the table in Supplementary Fig. 5c. Moving to experimental results in Supplementary Fig. 5d, we present the outcomes for the nine nonorthogonal channels. These results validate the effectiveness of our extended multiplexing method, showcasing the successful encoding and decoding processes across various wavelengths and polarizations.

Note 8. Design process for holographically generating elements in the periodic table

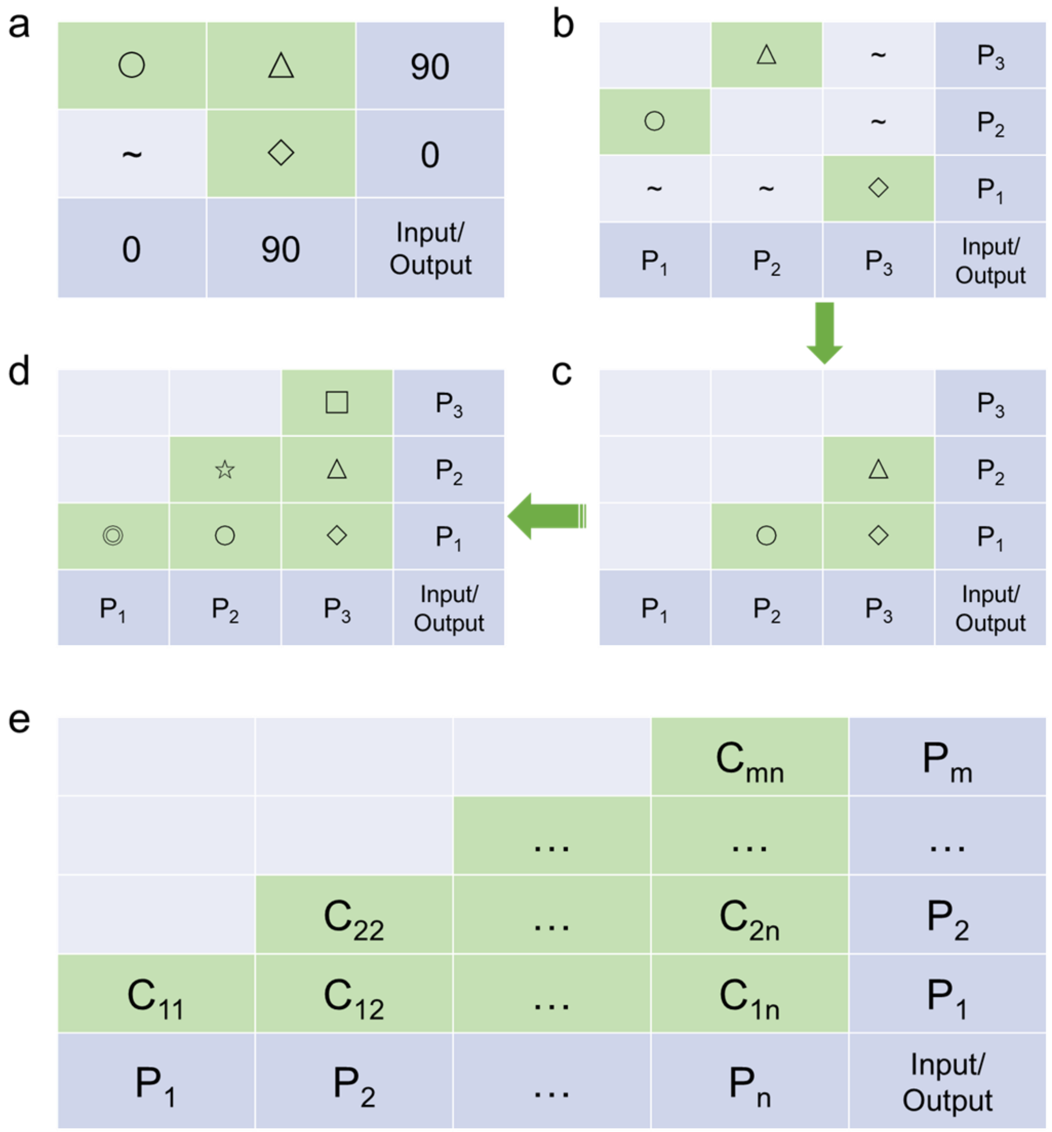

Supplementary Fig. 6. Design and evolution process of polarization multiplexing. a Traditional Jones matrix channels for multiplexing. **b** Design of three-channel nonorthogonal linear polarization multiplexing. **c** An equivalent conceptual representation of the design scheme in (b), highlighting the innovative concept. **d** Further extending the design. Three-channel nonorthogonal linear polarization multiplexing undergoes additional refinement. **e** Illustration of the multi-channel nonorthogonal polarization multiplexing design, showcasing the progression and sophistication of the multiplexing strategy.

Supplementary Fig. 6 chronicles the evolution of polarization multiplexing design, starting from traditional Jones matrix channels and advancing through various stages of refinement. The introduction of triple cyclic nonorthogonal linear polarization

channels represents a pivotal shift, with subsequent extensions and expansions demonstrating the ongoing development and complexity of the multiplexing approach.

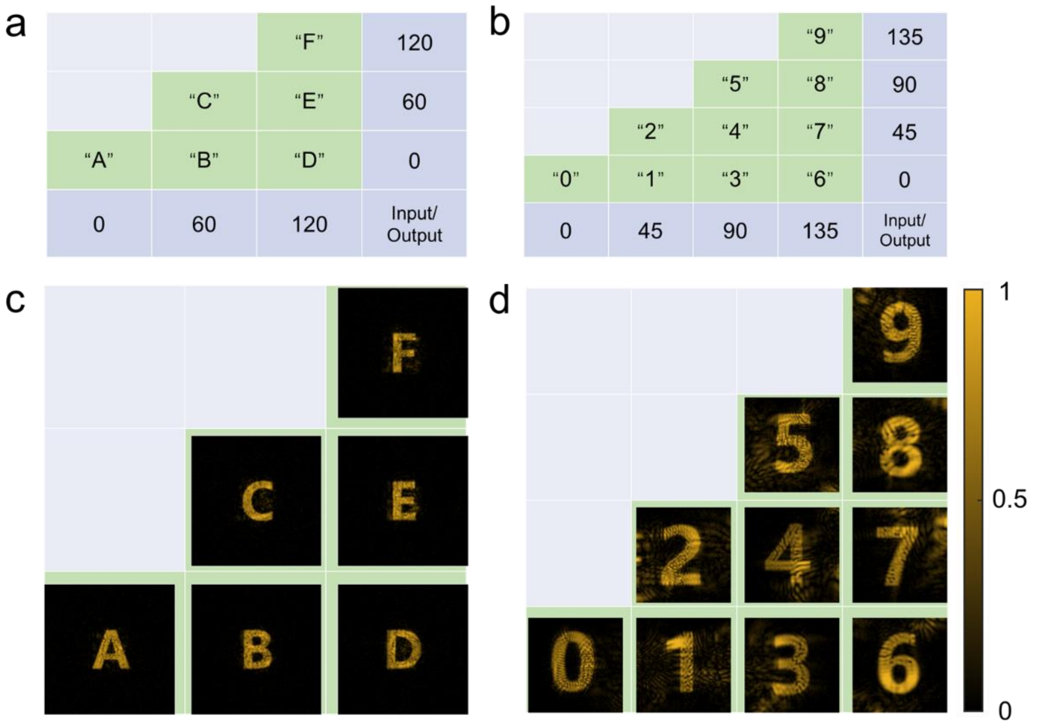

Supplementary Fig. 7. Evolution of nonorthogonal polarization holographic channels. a Design of a three-channel nonorthogonal linear polarization multiplexing. **b** Design of a four-channel nonorthogonal linear polarization multiplexing. **c** Scalar diffraction results from six channels designed at three polarization states. They provide insight into the holographic representation achieved through the evolving design. **d** Scalar diffraction results from ten channels designed at four polarizations. They showcase the increased sophistication and capacity of the holographic channels.

Supplementary Fig. 7 captures the progression of nonorthogonal polarization holographic channels, showcasing the evolution from triple cyclic designs to more intricate four-cycle structures. Scalar diffraction results based on the angular spectrum diffraction algorithm from both six and ten channels offer a visual representation of the holographic capabilities achieved at various stages of the design evolution. For more than three non-orthogonal polarization multiplexing, we optimized the metasurface by adopting the spatial dimension along the propagation direction. In the initial simulation, the metasurface dimensions are set at 225 μm × 225 μm. The diffraction distance for the main diagonal channel is established at 100 μm, while for the other three channels,

it is incrementally increased by 20 μm compared to the initial diffraction distance. This stepwise adjustment serves to showcase the versatility and adaptability of our nonorthogonal polarization-basis multiplexing strategy.

To underscore the scalability and robustness of our approach, we extend the simulation to encompass up to 55 channels, as elaborated in the main text. This expanded evolution provides a comprehensive demonstration of the strategy's effectiveness across a broader range of channels, emphasizing its potential for high-capacity information encoding and retrieval.

Note 9. Characterization of 55-channel holography

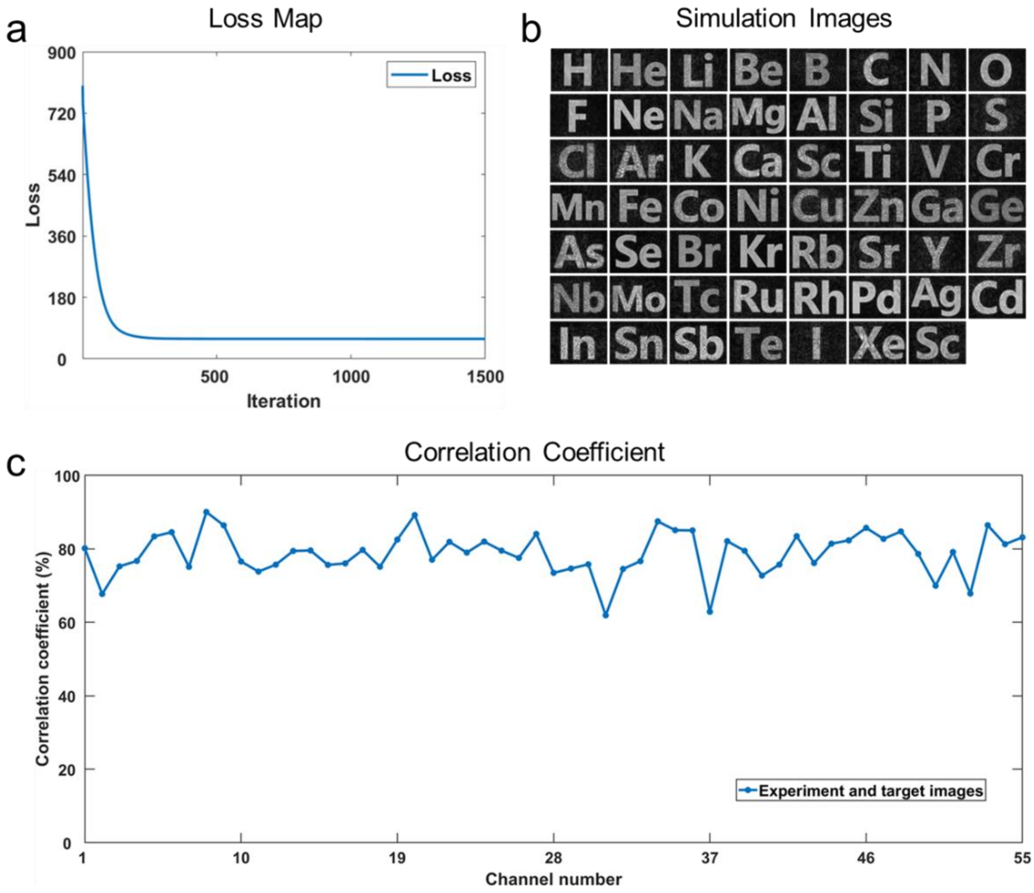

Supplementary Fig. 8. Characterization of 55-channel holography. a The loss map during the training process. **b** The optimized images after the training in the built neural network. **c** Correlation coefficient between experiment and target images in 55-channel holography.

Throughout the design process, we conducted 1500 training iterations. Supplementary Fig. 8a illustrates the loss map during the training process, where the loss decreases rapidly before stabilizing. Supplementary Fig. 8b displays the simulation diagram after the training, revealing a relatively clear image. The correlation coefficient between experiment and target images is presented as the curve in Supplementary Fig. 8c.

In order to quantitatively evaluate the results of the experiment, we calculated the correlation coefficient between the images obtained in the experiment and the target images, which correspond to the anti-diagonal elements in Fig. 5b. There is a very high degree of coincidence between the images. Moreover, due to the errors in manufacturing metasurfaces and the interference of cascaded optical elements in the optical path, the correlation coefficient in experiments is lower than that in simulations.

Note 10. Measured efficiencies of the 55 hologram channels

According to the holographic efficiency definition in the main text, the efficiencies of 55 nonorthogonal holograms are calculated and shown below. They have an average efficiency of 10%.

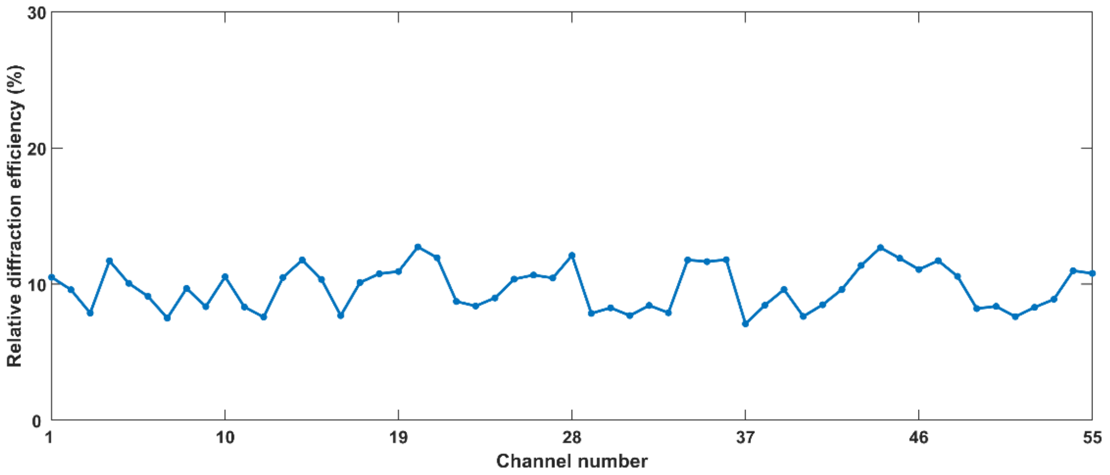

Supplementary Fig. 9. The measured efficiencies of 55 nonorthogonal holographic channels.
